# Nature and Treatment of Piles

**Published:** 1844-12

**Authors:** 


*Nature and Treatment of Piles.* By M. Lisfranc.—Hæmorrhoidal tumors are composed of a sort of fibrous tissue, in which only a few vessels are to be found when there is no congestion; and when this exists, however violent it may be, these vessels are never so numerous as in erectile tumors. On upwards of one thousand bodies, from which I removed the rectum, and in the numerous operations I have performed, I never as yet have met with a real erectile tumor in this region. This fact renders the prognosis less dangerous, and an operation not so indispensable. It may, therefore, be concluded—1. That though, without a doubt, veins more or less voluminous may be found in hæmorrhoidal tumors, still these last are not formed of varicose veins. 2. That their composition differs from that of erectile tumors.

Let us now examine the best mode of treatment for chronic hæmorrhoids. They cause little or no pain; if they protrude when the bowels are acted upon, the patient easily reduces them himself; they offer no ulcerations or indurations. In these cases most surgeons perform extirpation, without refleting on the danger which may follow this operation; for it must not be forgotten that it sometimes gives rise to very serious accidents. I never operate on similar occasions; and am convinced that a surgeon who cures without having recourse to the knife is far more useful than the most brilliant operator. I strive to diminish the size of the hæmorrhoidal tumors without employing the bistoury. A mild diet, gentle exercise, large or small venesections, as needed, produce generally an amelioration; when these fail, I direct on the part affected a shower-bath of Bareges water, or pure water, at the temperature of 68° F., and an injection into the interior of the rectum. By this method of treatment, if I do not succeed in obtaining a radical cure, at least I soothe the patient's sufferings. If you have not the means of administering a shower-bath, and the hæmorrhoids are protruded, by passing slightly over their surface the nitras argenti, not so as to cauterize, but merely to excite them, you will often succeed in obtaining their reduction; after which, in general, they do not re-appear. When the hæmorrhoidal tnmors are slightly ulcerated, I cauterize them with the nitrate of silver, or the acid nitrate of mercury. If the ulcerations are inveterate, and accompanied by induration of the surrounding parts, an operation is necessary; but that generally employed, viz., to seize the tumor with *Musieux pincers*, and to cut it off, may give rise to serious and even fatal hæmorrhage. The method I would advise in such cases does not present this danger, and is thus performed:—Two semi-lunar incisions, united by their extremities, must first be made on the tumor; this is then seized, so as to prevent the parts retracting as divided; the hæmorrhoid is next extirpated, not at once, as formerly, but by small incisions, so as to permit me to tie or twist the vessels as they are cut; finally, when it is nearly removed, I seize the pedicle between the forefinger and thumb, to be certain that no artery exists in its interior, and finish the operation slowly and carefully. A consecutive accident, very serious when it takes place, is *coarctatio recti.* In several cases Dupuytren was obliged to perform a second operation, in order to restore to the intestine its natural size, though he had employed all the necessary precautions to prevent this accident. During the first two or three days we must not introduce anything into the rectum; but as soon as the fears concerning the development of traumatic inflammation are over, we must have recourse to tents of large dimensions, and make the patient wear them constantly for at least three weeks, after which, for two or three months, he must introduce a large gum-elastic sound into the intestine every evening.—*Med. Times.—From Braithwaite's Retrospect.*

